# Intense versus standard regimens of intermittent occlusion therapy for unilateral moderate amblyopia in children: study protocol for a randomized controlled trial

**DOI:** 10.1186/s13063-020-04284-4

**Published:** 2020-04-28

**Authors:** Jingyun Wang, Ayesha Malik, Jing Jin, Yi Pang, Kelly Yin, Megan Allen, Adriana Grigorian, Brandy Scombordi, Joann Bailey, Saeed Aljohani, Katharine Funari, Ruth Shoge, Siva Meiyeppen, Jenny Myung, Ajay Soni, Daniel E. Neely

**Affiliations:** 1 Salus Univerisity Pennsylvania College of Optometry, 8360 Old York Rd, Elkins Park, PA 19027 USA; 2 Children’s Hospital of Philadelphia, Philadelphia, PA USA; 3 Nemours. Alfred I. duPont Hospital of Children, Wilmington, DE USA; 4 Wills Eye Hospital, Philadelphia, PA USA; 5 Illinois College of Optometry, Chicago, IL USA; 6 Arkansas Children’s Hospital, Little Rock, AR United States; 7 St. Christopher’s Hospital for Children, Philadelphia, PA USA; 8 Penn State Eye Center, Hershey, PA USA; 9 Glick Eye Institute, Department of Ophthalmology, Indiana University School of Medicine, Indianapolis, IN USA

**Keywords:** Amblyopia, Child, Intermittent occlusion therapy, Intense regimen, Randomized clinical trial, Visual acuity

## Abstract

**Background:**

We reported that in our previous study that wearing intermittent occlusion therapy glasses (IO-therapy) for 4 hours (h) was non-inferior to patching for 2 h in 3 to 8-year-old children with amblyopia. We hypothesize that an intense regimen of 12-h IO-therapy per day for 4 weeks could be as effective as the standard regimen of 4-h IO-therapy per day for 12 weeks in treating moderate amblyopia in 3 to 8-year-old children.

**Methods/Design:**

A total of 56 children between 3 and 8 years of age with amblyopia in association with anisometropia and/or strabismus will be enrolled. All participants will be prescribed IO-therapy glasses (Amblyz™), set at 30-s opaque/transparent intervals (i.e., occluded 50% of wear time). They will be randomized to receive the standard regimen for 12 weeks or the intense regimen for 4 weeks. Adherence to using the IO-therapy glasses will be objectively monitored in each participant by means of a microsensor dose monitor. The primary study objective is to compare the effectiveness of an intense regimen to a standard regimen of IO-therapy in 3 to 8-year-old children with moderate amblyopia. The secondary study objectives are to determine whether adherence differs between an intense regimen and a standard regimen of IO-therapy, and to determine the dose-response relationship of IO-therapy.

**Discussion:**

In addition to testing the effectiveness, this study will test for the first time the association between treatment adherence and the visual outcome of IO-therapy, which will enhance our understanding of the dose-response relationship of IO-therapy. If an intense regimen is shown to be effective, it would alter amblyopia treatment strategies and improve visual outcomes.

**Trial registration:**

ClinicalTrials.gov: NCT02767856. Registered on 10 May 2016.

## Background

Amblyopia, affecting 2–4% of all children, is the most common cause of monocular visual impairment in children [1, 2]. Traditional amblyopia treatment consists of penalization of the better eye using atropine drops or occlusion with an adhesive eye patch, which forces the amblyopic eye to have increased visual experience, while the stronger, fellow eye is pharmacologically blurred or occluded. In the USA, the current evidence-based recommendation is to prescribe 2  hours (h) of patching per day for moderate strabismic or anisometropic amblyopia [3–6]. The treatment may last from a couple of months up to years. Unfortunately, 20–25% of children do not respond to this patching treatment at all and 40% of children cannot achieve normal visual acuity [5]. Possible reasons include poor compliance, older age at start of treatment, insufficiently intense treatment regimen, or subclinical anatomicla or functional pathology [7]. Therefore, we continue to search for an alternative, child-friendly amblyopia treatment and protocol.

Recently, intermittent occlusion therapy (IO-therapy) with novel glasses utilizing liquid-crystal technology has presented an interesting alternative to existing treatment strategies [8–10]. Avoiding the use of adhesive patches, these IO-therapy glasses can be programmed to unilaterally alternate between opaque and transparent phases at 30-s intervals providing effective occlusion of the fellow eye 50% of the time they are worn. For example, the daily cumulative occlusion dose for 4 h of wearing IO-therapy glasses equals the occlusion dose for 2 h of patching. In addition, the optical correction can be incorporated into the IO-therapy glasses, which renders the occlusion less noticeable. Therefore, compared with adhesive patches, IO-therapy is more child-friendly [11]. We concluded from our previous study that the effectiveness of 4-h of wearing IO-therapy glasses with 50% occlusion is non-inferior to that of 2-h patching in 3–8-year-old children with amblyopia. Furthermore, the study supported the hypothesis that the cumulative amount of occlusion time is critical for the effectiveness of occlusion treatment in amblyopia; most of the children achieving their best visual acuity with a cumulative dose of 150–250 h [12].

If the cumulative amount of occlusion time is critical in occlusion treatment, it is reasonable to postulate that an intense regimen of IO-therapy may achieve faster vision improvement than is achievable with the standard regimen: Switching to a more intense regimen has been reported to be more effective in children with residual amblyopia, e.g., when the amblyopic eye stopped improving despite 2 h per day of the standard patching regimen, increasing the patching dosage to 6 h per day (three times the standard regimen dosage) resulted in further improvement compared with continuing the regimen for 2 h per day [13]. Moreover the adherence to patching decreases with treatment duration [14]. We recently found that adherence with IO-therapy also decreases with time [15]. An intense regimen with a faster outcome may improve adherence. When the child wears the glasses full time, it may require less effort from the parent to keep reminding the child to wear the device. Therefore, it is expected that such an intense regimen may improve adherence and lead to a better response to therapy.

To examine a comparison of the IO-therapy regimens, it is critical to monitor adherence objectively. Unfortunately, previous studies did not measure objective adherence using these IO-therapy glasses, limiting reliable assessment of the dose-response relationship of IO-therapy. Januschowski et al. attached a microsensor to common spectacles to monitor adherence [16]. This microsensor conveniently fits IO-therapy glasses; we have tested the feasibility of the sensor in monitoring adherence with IO-therapy glasses [15]. We will use this microsensor to monitor IO-therapy in this study.

Our working hypothesis is that an intense regimen of IO-therapy (three times the standard regimen dosage) with a shorter duration (one third of treatment duration) would be non-inferior to the standard regimen of IO-therapy with same cumulative amount of occlusion hours. For treating moderate amblyopia, 12 h per day (h/day) of IO-therapy for 4 weeks would not be inferior to 4 h/day of IO-therapy for 12 weeks. It can be simplified with a mathematical equation, where D represents day and WK represents weeks: 12 h/D x 4WK = 4 h/D x 12WK. To test this hypothesis, we designed a randomized controlled trial to compare the intense regimen with the standard regimen of IO-therapy for treating moderate amblyopia.

## Methods/design

### Objectives of the study

The primary study objective is to compare the effectiveness of an intense regimen with the standard regimen of IO-therapy in children ages 3 to ≤ 8 years with moderate amblyopia. The secondary study objectives are (1) to determine whether adherence differs between the intense regimen and the standard regimen of IO-therapy and (2) to determine the dose-response relationship in IO-therapy.

#### Ethical approval and conduct

This research protocol number HJW1604 and the informed consent forms were approved by the institutional review board (IRB) of Salus University. The clinical trial number is NCT02767856, registered on May 10, 2016 (www.clinicaltrials.gov). The Health Insurance Portability and Accountability Act will be observed during this study. Informed consent will be obtained from the participant’s parent or guardian (hereafter referred to as “parent”) by study investigators or coordinators; assent will also be obtained from participants 7–8 years of age. The IRB of Salus University, including interdisciplinary scientists, clinicians, and statisticians will monitor data safety annually.

#### Recruitment sites

**Fig. 1:**
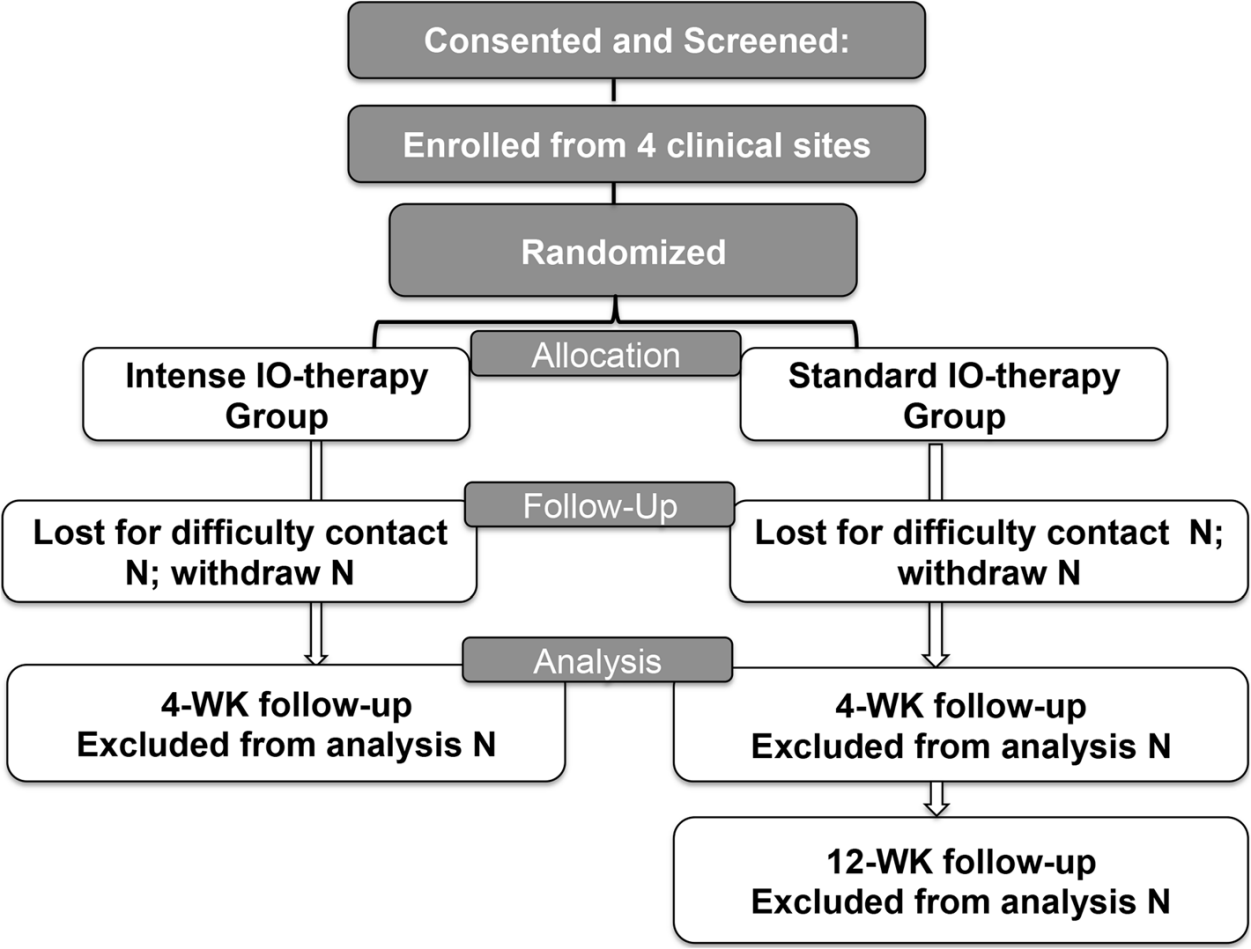
Study participant flow. IO-therapy, intermittent occlusion therapy

Four study sites in the USA will enroll potential study participants; they are The Eye Institute of Salus University, Philadelphia, PA (which is also the coordinating center); Illinois College of Optometry, Chicago, IL; Nemours. Alfred I. DuPont Hospital of Children, Wilmington, DE; and Arkansas Children’s Hospital, Little Rock, AR. They are all academic hospitals or clinics. A flyer will be used to advertise the study and the site principal investigators (PIs) and co-investigators will identify study participants. Participants may self-identify, contact the investigators, and join the study. The study has a randomized, parallel group, semi-masked design (examiners are masked), as depicted in Fig. 1.

**Fig. 2:**
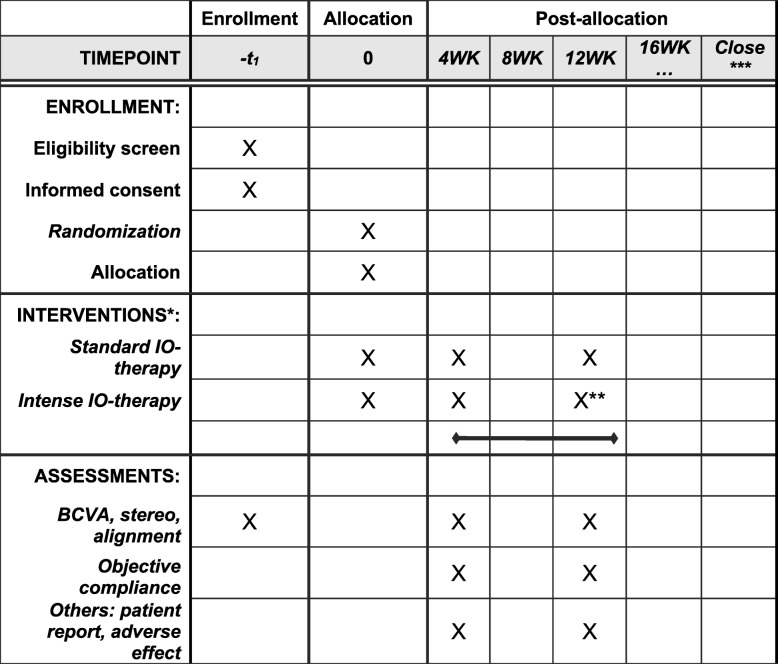
Standard protocol items: recommendation for interventional trials (SPIRIT) figure. IO-therapy, intermittent occlusion therapy

Eligibility testing includes measurement of visual acuity in both eyes using the standard Amblyopia Treatment Study single-surround HOTV letter protocol [17] and a routine comprehensive eye exam (comprehensive ocular examination and a full motility examination). Cycloplegic refraction is completed within 6 months.

The eligibility inclusion criteria are:

1. Age range from 3 to < 8 years, which is similar to the age range used in a previous Pediatric Eye Disease Investigator Group (PEDIG) study [5].
2. Unilateral amblyopia - best-corrected visual acuity (BCVA) of the amblyopic eye ranging from 20/40 to 20/80 [5]; interocular logMAR chart difference of at least two lines; BCVA in the sound eye of at least 20/40 or better.
3. Amblyopia associated with strabismus, anisometropia, or both.
4. Prior to the time of enrollment, the participant has worn optimal spectacle correction (if needed) for a minimum of 12 weeks or until stability of visual acuity was documented (no improvement in amblyopic eye visual acuity at two consecutive visits at least 4 weeks apart). Details of the protocol for refractive correction for moderate amblyopia followed guidelines of a previous PEDIG study [18].
5. The amblyopic eye was untreated by either patching or atropine for at least 6 months.
6. Gestational age > 34 weeks and birth weight > 1500 g.
7. Parents are willing to accept randomization, be contacted, and have access to a phone.

The exclusion criteria are:

1. The amblyopic eye has myopia worse than − 3.00 D spherical equivalent.
2. Prior intraocular or refractive surgery.
3. Ocular conditions that impact vision e.g. cornea scar.
4. Cognitive impairment that prohibits accurate data collection; Down’s syndrome or developmental delays.

#### Procedure

After confirming the enrollment criteria (Fig. 2), each participant will be randomized to one of two treatment groups:

- Standard group: 4-h daily wearing of IO-therapy glasses for 12 weeks
- Intense group: 12-h daily wearing of IO-therapy glasses for 4 weeks

The randomized group number for each participant is pre-sealed in an opaque envelope by a research assistant. All envelopes are kept in a box in sequence. When a participant is eligible for the study, we will have the participant open the envelope according to the sequence. If a participant is enrolled from a site outside of The Eye Institute of Salus University, the coordinator calls the central phone of the study manager, and the study manager will open the envelope in the sequence and inform the coordinator as to which group the participant has been randomized.

After randomization, each child will be provided a pair of IO-therapy glasses (Amblyz™ liquid crystal glasses, XPAND 3D Group, Limassol, Cyprus)*.* The glasses are set at 30-s opaque/transparent intervals for the non-amblyopic fellow eye. These glasses contain the child’s prescription and are rechargeable overnight. It often takes 7–14 days to prepare the child’s prescription spectacles to be fitted with the IO-therapy glasses and deliver them to the child.

To independently report adherence to wearing the IO-therapy glasses, all participants will be provided with a TheraMon microsensor (Hargelsberg, Austria) [16]. This inexpensive, commercially available, microsensor is attached to the temple arm of the IO-therapy glasses with commercial superglue and confers no additional risk to the wearer. Based on wearing events recorded using the microsensor, we will evaluate adherence at the scheduled clinic visits, data will be read and will provide a time history of episodes of wearing the IO-therapy glasses. A full description and a picture of the IO-therapy glasses attached with a microsensor and the mode of operation has been provided previously [15, 16]. The parent will be contacted by phone 1 week after initiation of the treatment, to answer any questions and to encourage compliance with treatment.

During the trial period, participants will not be allowed to receive other amblyopia treatments such as patching or atropine besides the prescribed hours of wearing the IO-therapy glasses or their own spectacles.

##### Primary outcome visit (both 4 ± 1 weeks and 12 ± 1 weeks)

After participants receive their glasses, the primary outcome visit will occur at 4 ± 1 weeks in the intense group and at 12 ± 1 weeks in the standard group. In addition, we ask the standard group to return at 4 ± 1 weeks for comparison with the intense Group.

Masked examiners, who do not know to which treatment group the participant is randomized, will perform both baseline and primary outcome tests. In addition to visual acuity, stereoacuity, and ocular alignment assessment, we also read microsensor data. Microsensor data will be logged by connecting the microsensors to the reading station via an antenna at a distance of 2–3 cm from the antenna. A USB cable will transfer the data to a PC; wearing times will be evaluated using the TheraMon® Software and compared to the wearing times protocol. The participant’s parent will be asked to comment on their child’s experiences with the IO-therapy glasses.

#### Adverse events

We monitor for potential major adverse events to include reverse amblyopia in the non-amblyopic eye (decrease of 2 lines in visual acuity), significant changes in ocular alignment (deviation changes of ≥ 10Δ), or any injury associated with the IO-therapy glasses or the microsensor. Any loss or possible breakage will be recorded. Study participants who lose or break the glasses will receive a new pair and continue the study.

##### Sample size calculation and randomization preparation

The sample size for this study was calculated based on a standard two-sided trial with a continuous outcome. The calculations assume 5% type I error with 80% power; the standard deviation of change from the baseline was 0.14, and the effective size difference was 0.12. Therefore, we anticipate that we will require 46 total participants (i.e., 23 in each group) [9]. Because the IO-therapy glasses are a novel device used to treat severe amblyopia, we are uncertain how many 3–8 year-old participants will drop out of the study. According to an average 15% dropout rate in previous amblyopia studies [6, 19], we have increased the sample size to 28 in each group to account for attrition. After collecting data from 28 participants, we will re-estimate the sample size based on updated attrition and standard deviation estimates [20].

With an online research randomization program (www.randomizer.org) of a permuted-block (*N* = 4) non-inferiority design, 56 participants will be randomized into the two treatment groups with 28 participants in each group. The allocation ratio is 1:1.

##### Outcomes and follow up

The primary outcome is the visual acuity change from the baseline in logMAR in the amblyopic eye as determined at the primary outcome visit. In the standard group, the primary outcome visit is at 12 ± 1 weeks; in the intense group, the primary outcome visit is at 4 ± 1 weeks. The study is continued if visual acuity in the amblyopic eye at the primary outcome visit has improved by at least one logMAR line; otherwise, the study is completed. We will also record parents’ feedback about the participants’ daily life experiences with the IO-therapy glasses.

If a participant meets the criteria to continue the study, the following steps will be observed: in the intense group, if equal vision is achieved in both eyes at the primary outcome visit, the participant will stop treatment for 8 weeks and return for a study visit; if vision is not equal in both eyes, treatment will continue for another 4 ± 1 weeks until there is no more improvement. In the standard group, participants return every 12 ± 1 week until there is no more improvement. Due to limited funding, there will be no more than three additional visits after the primary outcome visit in either group.

To protect participants’ confidentiality, all study-related procedures will be conducted in a private area - office or exam room - at a scheduled time; participant-related documents (consent forms and measurement reports) will be locked in a file cabinet, which only approved personnel can access. Data entry, storage, and management and protocol modifications follow Salus IRB instructions. The principal investigators and data analysts will access the final dataset. The principal investigators and co-investigators communicate periodically on study progress, updates, and study-relevant issues, such as protocol decision-making, manuscript writing, and publication. We will present at conferences and publish results. When the study is finished, we will communicate our findings to the parents. De-identified data tables will be shared by request after the primary outcome data have been published. A contact will be provided in the publication.

#### Adherence outcome

Adherence is defined as the percentage of hours the glasses are actually worn compared to the prescribed hours of wearing. Daily adherence is calculated, and general adherence is determined as the average of daily adherence in individuals. We will also evaluate the reasons for possible low adherence.

#### Analysis

Once a participant is randomized, the participant will be included in the analysis regardless of whether or not the assigned treatment is received. That is to follow the “intent-to-treat” design. Because the participant’s adherence is recorded by the microsensor, we will be able to better understand the dose-response relationship of IO-therapy through this study.

Descriptive statistics (mean and standard deviation) will be applied to the primary and secondary outcomes. The paired *t* test will be applied to analyze visual acuities before and after treatment in each group; the independent *t* test will be applied to compare visual acuity improvement in the two groups. Confidence intervals (CIs) for visual acuity improvement are reported in a non-inferiority manner [21]. The upper limit of a one-sided 97.5% CI will be computed on the treatment group difference in mean change in amblyopic-eye visual acuity, adjusting for baseline visual acuity, using analysis of covariance (ANCOVA).

The microsensor recording will provide data on the number of hours the glasses are worn each day. With total hours during treatment and improved visual acuity in the amblyopic eye, we can estimate the dose-response relationship for IO-therapy.

After the intervention type is considered, correlation coefficients will be calculated for the treatment response (improved visual acuity in the amblyopic eye) to the variables that are often suggested as important factors: (1) baseline visual acuity, (2) severity of amblyopia, and (3) participant age [22].

## Discussion

The trial protocol described herein aims to compare two regimens of IO-therapy glasses when treating children ages 3 to < 8 years who have unilateral amblyopia. We anticipate that the proposed study will provide the first data on the dose-response relationship of IO-therapy in treating moderate amblyopia. This study is designed to test the hypothesis: 12 h/D x 4WK = 4 h/D x 12WK.

Since visual acuity improvement in the amblyopic eye is not linear, it is possible that 4 h/day for 4 weeks is sufficient to be effective, and any additional hours are a waste of time. That is why we have another hypothesis: 12 h/D x 4WK = 4 h/D x 4WK. Therefore, we ask the standard group to return at 4 ± 1 weeks for comparison with the intense group.

Although cumulative occlusion hours matter in IO-therapy, treatment duration may also be important. Possibly the effectiveness of the intense regimen will not last as long as that of the standard regimen. It is advisable to add a longer follow-up period. Therefore, we need to test hypothesis: 12 h/D x 4WK + 0 h/D x 8WK = 4 h x 12WK. Ideally, we will have participants in the intense group return at 4 weeks and stop the treatment for 8 weeks, then return for assessment of the primary outcome. However, because this is a pilot study on the regimen, our IRB has concerns about the ethical issue of stopping treatment and requests us to continue to treat participants if they show improvement with IO-therapy. Therefore, in this phase, we are not able to test this hypothesis. We may test it in the near future based on the data from this project.

It is necessary to emphasize that the strength of this study is the use of microsensors. To avoid loss of the sensor or malfunctioning of the sensor, we advise participants to keep the glasses in the box indoors when they take them off.

According to the severity of amblyopia, PEDIG classified amblyopia into moderate amblyopia and severe amblyopia. Instead of 2 h/day of occlusion for moderate amblyopia, the dosage of 6 h/day of occlusion is recommended for severe amblyopia. In this study, we are only studying the regimen of treatment for moderate amblyopia. Therefore, the relevance of the results of this study is limited to the treatment of moderate amblyopia. In addition, after this study, we may investigate the sustainability of the IO-therapy effects in a large sample size.

### Trial status

Participant enrollment commenced in May 2016. The first participant was enrolled in October 2016, and the trial is scheduled to be completed by December 2020.

The trial was approved on 29 April 2016. It was amended on 27 June 2016, 29 June 2016, 13 September 2016, 27 September 2016, 15 December 2016, 17 February 2017, 22 February 2017, 19 April 2017 (continue review approval), 20 June 2017, 13 November 2017, 19 December 2017, 5 April 2018 (continue review approval), 27 August 2018, 18 December 2018, 6 March 2019, 9 April 2019, 10 April 2019 (continue review approval). It was amended 15 times and review was continued 3 times.

## Supplementary information

**Additional file 1.** Standard protocol items: recommendation for interventional trials (SPIRIT) 2013 checklist: recommended items to address in a clinical trial protocol and related documents.

## Data Availability

When the study is finished, a summary of results will be reported to www.clinicaltrials.gov.

## Abbreviations

BCVA: Best-corrected visual acuity
D: Day
h: Hour
IO-therapy: Intermittent occlusion therapy
IRB: Institutional review board
logMAR: Logarithm of the minimum angle of resolution
PEDIG: Pediatric Eye Disease Investigator Group
WK: Week
